# Verlängerung des Gesamtüberlebens durch die Impfung von autologen tumorlysatbeladenen dendritischen Zellen (DCVax-L) bei Patienten mit neu diagnostiziertem und rezidivierendem Glioblastom

**DOI:** 10.1007/s00066-023-02049-x

**Published:** 2023-01-20

**Authors:** Maximilian Y. Deng, Jürgen Debus, Laila König

**Affiliations:** 1 Klinik für Radioonkologie und Strahlentherapie, Universitätsklinikum Heidelberg, Im Neuenheimer Feld 400, 69120 Heidelberg, Deutschland; 2 Heidelberger Ionenstrahl-Therapiezentrum (HIT), Im Neuenheimer Feld 450, 69120 Heidelberg, Deutschland; 3 Nationales Centrum für Tumorerkrankungen (NCT) Heidelberg, Im Neuenheimer Feld 460, 69120 Heidelberg, Deutschland; 4 Klinische Kooperationseinheit Strahlentherapie, Deutsches Krebsforschungszentrum (DKFZ), Im Neuenheimer Feld 280, 69120 Heidelberg, Deutschland

## Hintergrund der Arbeit

Das Glioblastom stellt die häufigste hochgradig maligne, primäre Hirntumorentität dar. Trotz multimodaler Therapie aus neurochirurgischer Resektion, Radio- und Chemotherapie liegt das mediane Gesamtüberleben bei 15–17 Monaten. Insbesondere in der Rezidivsituation existieren keine etablierten (Standard‑)Therapien. Die vorliegende prospektive Phase-3-Studie untersucht, ob – verglichen mit der Radiochemotherapie – eine Verbesserung des Gesamtüberlebens für Patienten mit neu diagnostiziertem (nGBM) und rezidivierendem Glioblastom (rGBM) durch die zusätzliche Impftherapie von autologen tumorlysatbeladenen dendritischen Zellen (DCVax-L) erreicht werden kann.

## Studiendesign/Methode

Die multizentrische prospektive Phase-3-Studie rekrutierte im Zeitraum von August 2007 bis November 2015 insgesamt 331 Patienten an 94 Standorten in den USA, Kanada, dem Vereinigten Königreich und Deutschland. Nach erfolgtem „standard of care“ mittels Radiotherapie und simultaner Chemotherapie mit Temozolomid wurden die Patienten entweder in den Impftherapiearm mit DCVax‑L oder in den Placeboarm randomisiert (2:1). Das mediane Gesamtüberleben (mOS) der mit DCVax-L-behandelten Patienten mit nGBM wurde als primärer Endpunkt und das mOS der DCVax-L-Patienten mit rGBM nach Cross-over als sekundärer Endpunkt ausgewählt. Zum Vergleich diente jeweils eine externe Kontrollkohorte („external control population“ [ECP]) mit 1366 Patienten aus insgesamt 15 anderweitigen randomisierten Glioblastomstudien, die mittels Standardtherapie behandelt wurde.

## Ergebnisse

Insgesamt wurden 331 Patienten in die Studie eingeschlossen, wovon 232 der DCVax-L- und 99 der Placebogruppe zugewiesen wurden. Das mOS für die 232 mit DCVax‑L behandelten Patienten betrug 19,3 Monate nach Randomisierung im Vergleich zu 16,5 Monaten in der Gruppe der externen Kontrollpopulation (HR = 0,80; *p* = 0,002). Das mOS nach 48 Monaten betrug 15,7 % in der DCVax-L-Kohorte (vs. 9,9 %, Placebogruppe) sowie 13,0 % nach 60 Monaten (vs. 5,7 % in der Placebogruppe). Weitere 64 der 99 – initial aus der Placebogruppe stammenden – Patienten erhielten nach Rezidivdiagnose (rGBM) ebenfalls eine Therapie mit DCVax‑L im Sinne eines Cross-overs. Bei den mit DCVax‑L therapierten Patienten mit rGBM (*n* = 64) betrug das mOS 13,2 Monate ausgehend vom Zeitpunkt des Rezidivs im Vergleich zu 7,8 Monaten in der ECP (HR = 0,58; *p* < 0,001). Das mOS in der DCVax-L-Gruppe nach Rezidiv betrug nach 24 Monaten 20,7 % (vs. 9,6 %, ECP) und 11,1 % nach 30 Monaten (vs. 5,1 %, ECP). Zudem war ein Überlebensvorteil in den DCVax-L-Patienten mit nGBM festzustellen, die einen methylierten MGMT-Promotor aufwiesen (HR = 0,74; *p* = 0,03). Keiner der mit DCVax‑L behandelten Patienten wies eine Autoimmunreaktion auf. Bei insgesamt 2151 verabreichten Dosen wurden höhergradige Nebenwirkungen insgesamt 5‑mal festgestellt: Hirnödem (*n* = 3), schwere Nausea (*n* = 1) und Infektion der Lymphknoten (*n* = 1).

## Schlussfolgerung der Autoren

Die Ergebnisse der Phase-3-Studie zeigten auf, dass die Hinzunahme von DCVax‑L zur Standardtherapie mit einer klinisch relevanten und statistisch signifikanten Verbesserung des Gesamtüberlebens sowohl für neu diagnostizierte als auch für rezidivierende Glioblastompatienten verbunden ist, verglichen mit einer externen Kontrollkohorte (ECP) nach alleiniger Standardtherapie.

## Kommentar

Das Glioblastom gilt allgemein als immunologisch „kalte“ Tumorentität – die vergangenen prospektiven, randomisierten immuntherapeutischen Studien wiesen keinen ausschlaggebenden klinischen „benefit“ auf [1, 2]. Die vorliegende Studie beschreibt, dass durch die Anwendung eines Vakzins aus autologen tumorlysatbeladenen dendritischen Zellen (DCVax-L) – zusätzlich zur Standardtherapie – eine Verbesserung des Gesamtüberlebens (mOS) bei Patienten mit nGBM wie auch mit rGBM nach Cross-over erreicht werden kann [3]. Die Möglichkeit des Cross-overs im Studiendesgin wurde laut Autoren hierbei von der FDA gewünscht. Bemerkenswert ist, dass zum Zeitpunkt der Initiierung der DCVax-L-Studie das progressionsfreie Überleben (mPFS), und nicht das mediane Gesamtüberleben (mOS), als primärer Endpunkt herangezogen werden sollte [3, 4]. Die vorliegende Arbeit beschreibt, dass kein Unterschied hinsichtlich des mPFS zwischen der DCVax-Gruppe mit 6,2 Monaten und der Placebogruppe mit 7,6 Monaten zu verzeichnen war [3]. Somit wäre die Studie – wie sie initial geplant worden war – bzgl. des primären Endpunkts negativ ausgefallen (*p* = 0,47). Die Autoren erklären diesbezüglich, dass eine divergente Beurteilung der MRT-Bildgebungen in über 50 % der Fälle im zentralen Review der Radiologen vorzufinden gewesen sei. Die diffizile Unterscheidung zwischen realem Tumorprogress und Pseudoprogress sei ausschlaggebend für die Änderung des primären Endpunkts, die laut Autoren vor Entblindung erfolgte. In der Folge wurde mit dem mOS ein „harter“ Endpunkt ausgewählt.

Zudem wurden die Bewertungskriterien zur Definition eines realen Progresses beim Glioblastom nach Immuntherapie mehrfach aktualisiert: von den MacDonalds-Kriterien, wie sie initial in der vorliegenden Studie angewendet wurden, über die Response-Assessment-in-Neuro-Oncology-Kriterien (RANO) und „immunotherapy RANO“ (iRANO) bis hin zu den Modified-RANO-Kriterien [5–7]. Die Tatsache, dass es zu solch einer hohen Abweichung bei der Response-Beurteilung kam, adressiert ebenfalls eine gewisse Unsicherheit sowohl bei aktuell laufenden Studien als auch bei bereits publizierten Studien, die das PFS als Endpunkt gewählt haben [8–10].

Nach der Änderung des Studiendesigns wurde nun das Gesamtüberleben (mOS) der DCVax-L-Patienten mit neu diagnostiziertem Glioblastom (nGBM) als primärer Endpunkt ausgewählt, sowie das mOS der rezidivierenden Glioblastome (rGBM) – im Sinne eines Cross-overs – als sekundärer Endpunkt, die jeweils mit einer externen Kontrollpopulation („external control population“ [ECP]; *n* = 1366) verglichen wurden [11]. So wurde bereits harsche Kritik geäußert hinsichtlich der Vergleichbarkeit der DCVax-L-Kohorte und der ECP, da wesentliche Faktoren wie das Patientenalter, die Einnahme von Steroiden, der Performance-Status und der Resektionsgrad nicht ausreichend mitberücksichtigt worden seien [12]. Zudem wurden Patienten mit Zeichen einer „early disease progression“ nach Radiochemotherapie von der Studie ausgeschlossen, sodass hier ggf. eine Positivselektion zu beobachten ist [12].

## Fazit

Trotz der vielversprechenden Ergebnisse der vorliegenden Studie sollte ein möglicher Einzug der DCVax-L-Therapie in die Standardtherapie des Glioblastoms noch mit Zurückhaltung betrachtet werden. Eine klare Schlussfolgerung kann zum aktuellen Zeitpunkt – bei Anwendung des innovativen Studiendesigns und der damit einhergehenden nicht eindeutig zu interpretierenden Ergebnisse – noch nicht getroffen werden.

*Maximilian Y. Deng, Jürgen Debus,*

*Laila König, Heidelberg*
